# The Epidemiology of Transition into Adulthood of Rare Diseases Patients: Results from a Population-Based Registry

**DOI:** 10.3390/ijerph15102212

**Published:** 2018-10-10

**Authors:** Monica Mazzucato, Laura Visonà Dalla Pozza, Cinzia Minichiello, Silvia Manea, Sara Barbieri, Ema Toto, Andrea Vianello, Paola Facchin

**Affiliations:** 1 Rare Diseases Coordinating Center, Rare Diseases Registry, Veneto Region, 35100 Padua, Italy; laura.visonadallapozza@regione.veneto.it (L.V.D.P.); cinzia.minichiello@regione.veneto.it (C.M.); silvia.manea@regione.veneto.it (S.M.); sara.barbieri@regione.veneto.it (S.B.); ema.toto@regione.veneto.it (E.T.); 2 Department of Women’s and Children’s Health, University of Padua, 35100 Padua, Italy; andrea.vianello@unipd.it (A.V.); paola.facchin@unipd.it (P.F.)

**Keywords:** rare diseases, registry, epidemiology, transition

## Abstract

*Background*: Despite the fact that a considerable number of patients diagnosed with childhood-onset rare diseases (RD) survive into adulthood, limited information is available on the epidemiology of this phenomenon, which has a considerable impact both on patients’ care and on the health services. This study describes the epidemiology of transition in a population of RD patients, using data from the Veneto Region Rare Diseases Registry (VRRDR), a web-based registry monitoring since 2002 a consistent number of RD in a defined area (4.9 million inhabitants). *Methods:* Longitudinal cohorts of patients born in the years 1988 to 1998 and enrolled in the VRRDR in their paediatric age were identified. Data referred to this group of patients, experiencing transition from paediatric to adult age during the years 2006–2016, are presented. *Results*: 2153 RD patients (44.1% females and 55.9% males) passed from childhood to adulthood in the study period, corresponding to a 3-fold increase from 2006 to 2016. The majority of these patients was affected by congenital anomalies (32.0%), by hematologic diseases (15.9%), eye disorders (12.1%) and neoplasms (7.9%). RD patients who experienced transition from paediatric age to adulthood represent the 9.2% of adult patients enrolled in the Registry at 31 December 2016. *Conclusions:* We described a subset of RD young adults experiencing transition into adulthood. The data reported can be considered as minimum values for estimating the size of this increasing population presenting specific transition needs. These figures are valuable for clinicians, patients and health planners. Public policy interventions are needed in order to promote dedicated care transition pathways in the broader framework of health policies devoted to RD.

## 1. Introduction

Rare diseases (RD), defined in Europe as those with a prevalence of less than one per 2000 inhabitants, are a numerous group of diseases, with often a neonatal/paediatric onset and a chronic, progressive disabling course [1]. They represent a very complex medical issue, requiring life-long, highly specialized, coordinated care. Their public health relevance relies on the fact that, despite the number of patients affected by a specific RD can be very limited, the global number of patients living with a RD is considerable. Fortunately, in recent years, advances in medicine and access to new effective treatments have improved the prognosis of many of these conditions. Therefore, a growing number of paediatric patients with previously fatal rare diseases now survive into adulthood. Despite this success, several studies have highlighted that without adequate support, adolescents transitioning from the paediatric to the adult health care system are at increased risk of poor health outcomes [2,3,4,5,6,7,8,9]. Specific interventions have been put in place to support patients during this process, especially adolescents with complex medical conditions. The term “transitional care” entered in use to describe “*the purposeful, planned movement of adolescents and young adults with chronic physical and medical conditions from child-centred to adult-oriented healthcare systems*” [10]. 

The growing attention on this topic is the result not only of the increasing proportion of paediatric patients experiencing transition into adulthood, but also of the potential additional burden that this phase poses on the individual, the family and the health care services. Some aspects of complexity are intrinsic, as transition occurs at the same time of other important changes in life, per se challenging, related to the physical, psychological, and relational domains [11,12,13]. 

Patients with long-term conditions, and rare diseases patients in particular, face even greater challenges, since, in addition, they can experience modifications in their care needs and often change providers. Several studies have explored the consequences that an unsuccessful care transition can cause. These include, but are not limited to, severe health outcomes due to scarce follow-up compliance and treatment and increased risk of emergency admissions and hospitalization due to acute complications [14,15,16,17]. Furthermore, young adults aged 18 years and over constituted almost 5% of discharges from 10 U.S. paediatric hospitals, a datum that can be expression of a difficult transition process for youth adults with long-term chronic diseases [18]. 

Initially, the literature on this topic has been mainly focused on chronic common conditions such as diabetes and asthma, but now transition is acknowledged as a priority across all long-term conditions, both frequent and rare ones [19]. 

Many national plans for rare diseases address the issue of transition [20,21,22,23,24]. Furthermore, the care transition has been identified as an important area of activity within the European Reference Networks, recently established in application of the Directive 2011/24/EU of the European Parliament and of the Council [25,26,27]. Whilst the attention deserved to this topic is increasing, we still face major knowledge gaps. A variety of interventions has been set up to prevent negative outcomes of unsuccessful transition, but the evaluation of the impact of these interventions is affected by limitations, as outlined by a recent systematic review [28]. 

Whilst some recent studies have described the epidemiology of rare diseases in general and their impact [29,30], we face a general lack of epidemiological data referred to rare diseases patients diagnosed with a RD in their childhood and reaching the adult age. The aim of the present study is to describe the epidemiology of transition from childhood to adulthood in rare diseases patients, using data from the Veneto Region Rare Diseases Registry (VRRDR), a web-based registry monitoring since 2002 a consistent number of rare diseases in a defined population (nearly 4.9 million inhabitants).

## 2. Materials and Methods 

### 2.1. RD Framework 

In Italy, persons with one of the RD included in the national RD list [31] can obtain free of charge diagnostic procedures, follow-up visits, drugs, medical devices, provided they have been diagnosed and certified by Centers of expertise, officially labelled at regional/interregional level. 

The list contains 331 single diseases or groups of disorders divided into 14 nosological categories, based on the ICD9-CM. For the groups, only some examples of the included diseases are provided in the list (see Supplementary Materials). The identification of all RD included into groups, whose affected patients are eligible for benefits, was performed by the medical staff of the registry. This work is subject to continuous updating. Nearly 3000 RD entities have been identified, which are monitored by the RD registry since 2002. Each monitored disease is described by codes used in international classifications (i.e., ICD9-CM, ICD-10, MIM and Orphacodes). 

The list of RD has been updated in 2017 [32]. The coverage of the list, compared to the Orphanet one, has consequently increased, due to the growing number of RD entities included. Nevertheless, some diseases considered as rare in Europe, i.e., cystic fibrosis and many rare tumors, are not present in the Italian updated RD list and are monitored through distinct dedicated registries. Excluding these entities, the RD monitored by the registry represent 58% of all the rare diseases included in the Orphanet database [33]. For the present study, referred to the years 2006–2016, the list of monitored RD in effect during the study period was considered (Supplementary Materials). 

### 2.2. The RD Care Network 

Veneto Region identified Centers of Expertise for groups of RD in 2002, in accordance to the national health policy on rare diseases. Since 2002 the Veneto region RD care network, composed of public hospitals, has undergone four official revision processes. These evaluations have been based on the Centers’ activity, demonstrated by the registry’s data, and on the analyses of other independent data sources, i.e., hospital discharge records, rehabilitation services’ data, drugs’ registries, etc. Different clinical wards, either within the same hospital or in different hospitals, constitute the RD Centers of Expertise, labelled for groups of RD. Each Center is composed by both paediatric and adult clinical wards. Rehabilitation services are fully part of the care network. Of note, regional hospitals of the RD care network are currently full members of 18 out of 24 European reference networks (ERNs) [34]. 

### 2.3. The RD Registry 

The Ministerial Decree n. 279/2001 envisaged the establishment of a RD Registry at the National Institute of Health [35]. Due to the decentralized health system organization, the set-up of regional/interregional registries was in charge to Regions. Regions have set up registries according to different modalities, mainly within regional health information systems. The design and functioning of the Veneto region RD Registry have been already described in detail [29]. 

In the Veneto region, all the health care providers involved in RD patients’ care use a common Information System (IS), which is intended not only as an epidemiological registry, but as a tool fully supporting the RD patients’ care pathways and the provision of services to them. The IS connects through a protected network (Regional Health Network Intranet) the labelled RD Centers of expertise, located at hospital level, all the local health authorities, the territorial services and all the pharmaceutical services of the Region. More than 2017 users access the system, of whom 935 are clinicians working in RD Centers. 

According to the national legislation, a patient with a clinical suspicion of a rare disease has to be referred to a specific Center of the RD network in order to have a complete assessment, which is free of charge, only if performed in an officially labelled RD Center. Diagnosis’ recording implies the access to specific benefits for patients, such as drugs and/or medical devices listed in the care plan, defined by the Center of expertise. The system collects socio-demographic and clinical data of RD patients, respecting the National regulation on data protection. Specific modules have been progressively developed starting from the diagnosis definition. The IS allows the online prescriptions of treatments (i.e., drugs, para-pharmaceuticals, galenicals, dietetic products, medical devices, etc.) and the reporting of possible related adverse events. Information on clinical data, specific per group of RD, is collected in the IS according to a hierarchical logic. 4957 signs, 294 symptoms, 13,861 co-morbidities, 278 impairments and 18,201 localizations constitute the thesaurus at the basis of the clinical patient summary within the IS. 

### 2.4. Setting and Subjects

The population monitored includes all residents in the Veneto Region, i.e., a population of 4,907,529 as at 2016 (source: Italian National Institute of Statistics). Eligible cases in the VRRDR are all patients diagnosed with one of the rare diseases included in the Italian RD list. Clinicians working in the RD Centers of expertise of Veneto Region input data of patients diagnosed and followed up by them. Health professionals working in the regional health districts input data of RD patients diagnosed and followed up by Centers of Expertise outside the regional area being monitored. In this way, a comprehensive coverage of the Veneto’s resident population is achieved. Cross-referencing with other data sources available at regional level is regularly performed. These sources include the registry of hospital discharge records, the birth registry, the records of outpatient rehabilitation services, and the death registry. 

For this retrospective cohort study, we have considered data referred to Veneto region residents. The limit of 18 years was considered as a cut-off for the transition from paediatric care to adulthood care, as, by regulation, youth must transfer to adult care by this age. Longitudinal cohorts of patients born in the years 1988 to 1998 and enrolled in the VRRDR when they were under 18 years of age were identified (transition cohorts). Data referred to this sub-group of patients, experiencing transition from paediatric to adult age during the years 2006ߝ2016, are presented. 

Statistical analyses were performed using the SAS package, rel. 9.4 (SAS Institute Inc., Cary, NC, USA). Tables and figures were produced using Microsoft Excel, Office 2013 (Microsoft (Redmon, WA, USA).

## 3. Results

At 31 December 2016, there were 29,826 individuals residing in Veneto region, diagnosed with one of the rare diseases listed in the Italian Law (see Supplementary Materials) and registered in the VRRDR. 

**Figure 1:**
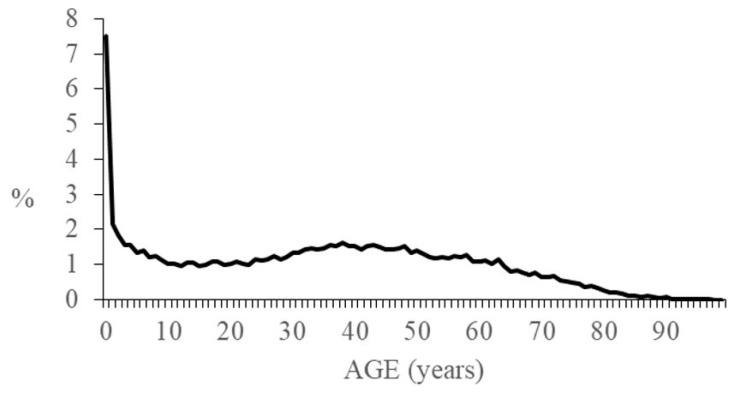
Distribution per age at diagnosis in patients with rare diseases. Veneto Region Rare Diseases registry, 2016.

Of the 29,826 patients who lived in the study area, 51.6% were male and 48.4% female. The mean age of enrolled patients was 34.2 years old. The distribution per age at diagnosis is presented in Figure 1. Patients receiving a diagnosis under 18 years of age are 8667 (29.1%): 7767 (26.1%) are under 14 years of age and 900 (3.0%) are aged 15–17 years. In this group of patients, mean age at diagnosis is 5.8 years, whilst median age at diagnosis is 4 years. A majority of males can be observed: 57.2% versus 42.8% females. 

We have considered all patients potentially experiencing transition in the study period because they were aged 15 to 17 years. Overall, during the study period, these patients were 4291. Of them, nearly half are patients diagnosed and enrolled in the registry when they were under 14 years of age, and 17.3% when they were aged 15–17 years. Nearly one out of three are patients enrolled in the VRRDR after the age of 18 years. 

**Figure 2:**
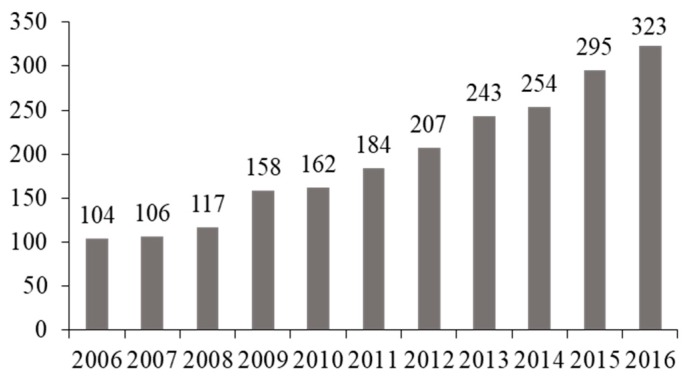
Distribution of RD patients per transition cohort (2006–2016).

Considering the limit of 18 years as a cut-off for the transition into adulthood, Figure 2 presents the distribution of RD patients per transition cohort. 

Overall, 2153 rare diseases patients, (44.1% females and 55.9% males) passed from childhood to adulthood in the study period. The number of patients experiencing transition steadily increased, corresponding to a 3-fold increase in the 11 years considered. The cohorts’ comparison did not highlight main differences in terms of socio-demographic data. Regarding RD diagnoses, we observed only a slight increase of patients diagnosed with congenital anomalies and rare eye diseases during the years, probably due to better case ascertainment, thanks to the evolution and availability of new genetic diagnostic techniques.

Patients who experienced transition from paediatric age to adulthood in the study period represented nearly the 9.2% of all the adult patients enrolled in the Registry (n = 23,496). The raw prevalence rate in the paediatric population residing in the study area (n = 809,344 Source: Italian National Institute of Statistics, 31/12/2016) is 26.6 per 10,000. During the study period, 26 patients, diagnosed with a RD in their childhood, did not survive into adulthood.

As shown in Table 1, nearly 1 out of 3 patients is affected by a congenital anomaly (32.0%). The remaining RD patients experiencing transition into adulthood are diagnosed with a rare hematologic disease (15.9%), a rare eye disease (12.1%), a rare tumor (7.9%) or an inborn error of metabolism (7.7%). 

This distribution differs from the one of RD paediatric and adult patients enrolled in the VRRDR at 31.12.2016 (Table 2). In the pediatric sub-group of RD patients, a greater proportion is affected by a congenital anomaly (42.9%). 17.2% of paediatric patients are affected by rare hematologic diseases, followed by rare eye diseases (6.2%) and inborn errors of metabolism (7.8%). In adult patients the most represented group of RD are eye diseases (19.5%). Interestingly, diseases manifesting at birth or in the early paediatric period, as congenital anomalies (8.7%) and inborn errors of metabolism (9.1%) accounted for a non-negligible proportion of rare disease cases in adult population.

**Figure 3:**
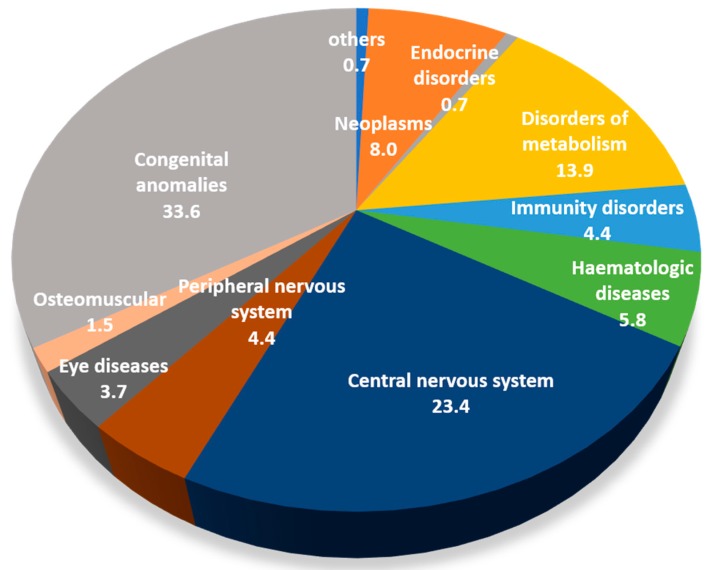
Percentage distribution per nosological group (ICD9-CM) of RD patients whose death occurred in paediatric age. Veneto Region rare diseases registry; 2006–2016.

During the study period, 157 deaths occurred in paediatric patients. Mortality data referred to this subset of patients are presented in Figure 3. 

Congenital anomalies are responsible for 33.6% of deaths registered in the study period, followed by central nervous system diseases (23.4%) and by inborn errors of metabolism (13.9%). While these three groups of rare conditions describe more than half of all the prevalent cases among paediatric patients (56.8%), they were responsible for two thirds of the deaths in children and adolescents occurred during the study period (75.3%).

## 4. Discussion

Several studies tackle transition issues focusing on chronic conditions either in general or on specific diseases, rare or more common ones. This consistent body of literature addresses factors influencing this multifaceted dynamic process and reviewed pilot projects and programs, focusing on determinants of a successful transition of care to adult providers. 

From a public health point of view, it is of paramount importance to describe not only the burden that an unsuccessful transition can cause on patients, families and health care systems, but to estimate the magnitude of this phenomenon at population level. Some data are available, but they are referred to children with chronic conditions and to children with special health care needs (SHCN), defined as “those who have or are at increased risk for a chronic physical, developmental, behavioral, or emotional condition and who also require health and related services of a type or amount beyond that required by children generally” [35]. It has been estimated that 90% of children with chronic conditions in the US survive into adulthood and that approximately 13% of youth (aged 0–17 years) have special health care needs. The percentages are expected to increase as survival of these children fortunately continues to improve [36,37].

Both these two categories, children with chronic conditions and children with SHCN, encompass, but do not coincide with RD paediatric patients. Some studies have addressed the peculiarities of the transition process in RD patients. These studies highlight that the “one size fits all” model for transition does not apply to these patients, who experience specific hurdles in the transition process. 

Whilst the attention on issues surrounding the care transition in RD patients is growing, we face a general lack of epidemiological data quantifying the impact of this phenomenon in RD patients. Few studies have provided the first estimates of the collective impact of RD at population level. The same studies have described the reasons at the basis of the difficulty in providing epidemiological figures referred to a consistent number of unrelated RD [29,30]. These difficulties are even greater when we want to assess which is the proportion of patients with RD of childhood onset who experience the transition into adulthood, as a population-based approach is needed, combined with a sufficiently long period of observation. 

To our knowledge, this is the first study proving epidemiologic data quantifying the magnitude of this phenomenon in RD patients, using data from a population-based registry, ongoing since 2002 and monitoring a consistent number of RD entities. The figures reported provide an estimate of the proportion of young adults with childhood-onset rare diseases in a given geographic region moving to adulthood oriented care. 

Before discussing our findings, we must address some limitations of the study. First, the list of rare diseases monitored by the described registry covers a considerable number of RD, but not all the diseases entities that can be defined as rare, according to the prevalence cut-off adopted in Europe [38]. This limitation has been partially overcome in 2017, with the official update of the Italian RD list, which mitigated the under-representation of specific groups of diseases, such as respiratory disorders and renal diseases, in the original list. For the present study, we did not considered the updated list, as the presented data are referred to the period 2006–2016, during which the previous list was in force. Nevertheless, the global amount of RD monitored at that time by the considered registry was consistent, corresponding to more than 3000 entities. 

Another criticism is that we could have underestimated the number of adult survivors of childhood RD, due to under-ascertainment of cases or loss to follow-up. Regarding the first aspect, the registry supports the functioning of the RD care network, established since 2002, and based on Centers of expertise, selected on an official basis. This turns into a high level of expertise in diagnosis and care of RD patients, demonstrated by the attraction rate of the regional network, caring for up to 17% of patients coming from other Italian regions, and by the participation of many regional Centers to the European Reference Networks (ERNs). Furthermore, the population coverage achieved by the registry is supposed to be high, as the registry is established by law, and RD patients’ enrollment is linked to the issue of special benefits, on the basis of national and regional regulations, such as free of charge provision of diagnostic assessment, drugs, including high costly treatments, and medical devices. 

Another limitation of the study is that we could not explore to what extent the growing number of RD patients in transition was the effect of an increased diagnostic ability, of a better referral to Centers of expertise of suspected RD patients, of an increase in survival due to better response to therapies or access to innovative ones. This area of research deserve certainly future investigations, using complementary additional data sources. 

Another potential limit is that we have considered the age of 18, as the age at which patients experience transition to adult-oriented care providers. We must acknowledge that this reflects the prevailing situation in the described setting and cannot be generalized to other countries in which transition may occur at age 16 or at older ages [39,40]. 

Furthermore, we did not explore the transition experiences of RD patients diagnosed in their childhood, especially in terms of which are the factors that influence the ability to carry out a satisfactory and efficient care transition. Despite these potential pitfalls, we believe this study shades light on the growing phenomenon of RD patients moving from paediatric age to adulthood.

The identified cohorts represent the 9.2% of the adult patients diagnosed with a RD in the study area. Considering the paediatric population resident in the study area, the raw prevalence of RD patients experiencing transition into adulthood is 26.6 per 10,000. Considering the above reported limitations of the study, this should be considered as a minimum value describing the size of this subset of patients. Furthermore, this study shows which is the relative contribution of different nosological groups in the population of RD patients experiencing transition. Patients affected by congenital anomalies constitute the most represented group. This is in accordance with previous studies that have highlighted an increased survival into adulthood of these patients [41,42]. Hematological diseases (15.9%) were the second most represented group of patients contributing to the transition population. It has to be reported that a high prevalence of thalassemia and sickle cell disease exists in the area under study, due to endemic diffusion and high immigration rates, respectively [43,44]. Another consistent group of patients is represented by patients affected by inborn errors of metabolism, which represent a consistent proportion of adult patients living with a RD having a childhood onset. Which will be the impact of expanded newborn screening on the future growth of this subset of RD patients surviving into adulthood is an area that deserves particular research attention [45]. 

It is interesting to notice that studies exploring factors affecting the transition process or providing specific transition guidelines are available for all the above mentioned groups of RD, but none of them provides data on the global number of patients potentially experiencing transition into adulthood [46,47,48,49,50,51]. When available, these figures are scattered, either because referred to a single Center experience and/or to a single rare disease [52,53]. 

Our data support the need for a global rather than a piecemeal approach, highlighting the transition issue as a pivotal area for the development of specific health policies, taking into account the specificities of RD. The transition process assumes peculiar aspects of complexity for RD patients. First, RD are often multisystemic, requiring coordinated care that involves many different specialists. Whilst this multidisciplinary approach is often provided within the same paediatric care setting, it requires a more complex coordination effort when it involves specialists working in different medical Centers for adult patients. Second, for some rare diseases, previously causing early mortality, the expertise is scattered and less common to find in the adult care setting. Furthermore, the natural history of these diseases in adulthood is only partially known, and consequently the knowledge of their care management is limited as well, due to paucity of existing guidelines and follow-up data. Third, paediatric RD patients moving to adult care can present with cognitive impairments and other co-morbidities, making the process of transition particularly complex. For all these reasons, the issue of transition is perceived as critical by both RD patients and healthcare professionals taking care of them [54].

We would like to focus on the fact that, when appropriately tackled from a health planning point of view, the issue of transition can represent a valuable opportunity to transform the entire system of care, starting from a population in need of highly integrated services [55]. The continuity of care and collaboration between paediatric and adult care providers is a strategic factor for a successful transition. Evidence supports the idea that paediatric and adult-oriented medicine represent two different expressions of medical knowledge [56]. This is amplified when considering RD. In many cases, children and adults, despite having the same RD diagnosis, can present with completely different phenotypes and related health needs. No successful transition can occur in these cases without the sharing of information between paediatric and adult providers caring for the same patients across time [56]. 

The described registry, in which clinical data and treatments’ prescriptions are shared among health professionals working in different settings, caring for the same RD patients, especially those in transition, can be considered a valuable tool to aid in this process. Moreover, especially when considering the growing number of RD patients that will experience transition, we advocate for the removal of administrative barriers, that potentially can affect patients’ care pathways. We encourage the adoption of reimbursement procedures, and thus resources’ allocation processes, based on care pathways rather than on the activity of clinical wards, separately considered. Furthermore, the identification of dedicated settings within hospitals where RD Centers of expertise are located, in which the multidisciplinary care can effectively occur, can be another action to undertake in order to promote effective care coordination and, thus, a successful care transition. 

## 5. Conclusions

The number of children and youth with rare diseases surviving into adulthood is increasing and represents a non-negligible portion of the whole RD population. This subset of patients presents specific transition needs, as the process of referral from the paediatric care setting to the adult healthcare system can be particularly challenging for them. As unsuccessful transition can be associated with particularly severe outcomes for this vulnerable sub-group of patients, health policy decisions addressing the specificities of the passage from paediatric medicine to adult medicine for RD patients should be strongly advocated, as they can reduce potential long-term adverse health outcomes, together with human and social costs. The development of care pathways and the use of emerging health information technology connecting paediatric and adult care providers should be promoted, in order to properly address the complex health care needs of this special population and guarantee the successful management of the transition process. 

## Figures and Tables

**Table 1:** Percentage distribution per nosological group (ICD-CM) of patients experiencing transition. Veneto region rare diseases registry; 2006–2016.

GROUPS OF DISEASES (ICD9-CM)	
Infectious and parasitic diseases	0.0
Neoplasms	7.9
Endocrine disorders	3.6
Disorders of metabolism	7.7
Immunity disorders	4.1
Diseases of the blood and blood-forming organs	15.9
Central nervous system disorders	2.6
Peripheral nervous system disorders	4.5
Disorders of the eye and adnexa	12.1
Diseases of the circulatory system	3.9
Diseases of the digestive system	0.7
Diseases of the genitourinary system	0.1
Diseases of the skin and subcutaneous tissue	0.7
Diseases of the musculoskeletal system and connective tissue	4.0
Congenital anomalies	32.0
Certain conditions originating in the perinatal period	0.3

**Table 2:** Percentage distribution per nosological group (ICD-CM) of pediatric and adult RD patients. Veneto region rare diseases registry; 2016.

	Patients	Patients
GROUPS OF DISEASES (ICD9-CM)	(0–17 Years)	(=>18 Years)
Infectious and parasitic diseases	0.0	0.4
Neoplasms	6.6	2.8
Endocrine disorders	2.4	2.0
Disorders of metabolism	7.8	9.1
Immunity disorders	3.4	4.8
Diseases of the blood and blood-forming organs	17.2	11.7
Central nervous system disorders	3.0	10.3
Peripheral nervous system disorders	3.1	6.9
Disorders of the eye and adnexa	6.2	19.5
Diseases of the circulatory system	3.7	4.1
Diseases of the digestive system	0.4	2.7
Diseases of the genitourinary system	0.1	1.2
Diseases of the skin and subcutaneous tissue	0.7	5.5
Diseases of the musculoskeletal system and connective tissue	2.2	10.1
Congenital anomalies	42.9	8.7
Certain conditions originating in the perinatal period	0.2	0.0

## Supplementary Materials

The following are available online at http://www.mdpi.com/1660-4601/15/10/2212/s1. List of rare diseases monitored by the Veneto Region’s rare diseases registry. Adapted from the Italian Ministerial Decree on rare diseases—Annex 1 Reference [31].
